# The role of aetiology in cardiac manifestations of chronic kidney disease: the CPH-CKD ECHO study

**DOI:** 10.1007/s10554-024-03092-0

**Published:** 2024-04-30

**Authors:** Jacob Christensen, Nino Emanuel Landler, Flemming Javier Olsen, Ida Maria Hjelm Sørensen, Sasha Saurbrey Bjergfelt, Ellen Linnea Freese Ballegaard, Bo Feldt-Rasmussen, Ditte Hansen, Anne-Lise Kamper, Christina Christoffersen, Susanne Bro, Tor Biering-Sørensen

**Affiliations:** 1 Department of Cardiology, Copenhagen University Hospital - Herlev and Gentofte, Copenhagen, Denmark; 2 Center for Translational Cardiology and Pragmatic Randomized Trials, Department of Cardiology, Copenhagen University Hospital - Herlev and Gentofte, Copenhagen, Denmark, University of Copenhagen, Niels Andersens Vej 65, 2900 Hellerup, Copenhagen, Denmark; 3 Department of Nephrology, Copenhagen University Hospital - Rigshospitalet, Copenhagen, Denmark; 4 Department of Nephrology, Copenhagen University Hospital - Herlev and Gentofte, Copenhagen, Denmark; 5 Department of Clinical Biochemistry, Copenhagen University Hospital - Rigshospitalet, Copenhagen, Denmark; 6 Department of Biomedical Sciences, Faculty of Health and Medical Sciences, University of Copenhagen, Copenhagen, Denmark; 7 Department of Clinical Medicine, Faculty of Health and Medical Sciences, University of Copenhagen, Copenhagen, Denmark

**Keywords:** CKD, Cardiovascular research, Echocardiography, Aetiology

## Abstract

**Purpose:**

We investigated the associations between cardiac parameters and aetiologies of CKD in an exploratory study.

**Methods:**

The study population consisted of 883 participants, 174 controls and 709 patients with aetiologies of CKD including diabetic nephropathy/renovascular KD in diabetes mellitus, hypertensive/renovascular nephropathy, tubulointerstitial nephritis, glomerulonephritis/vasculitis, polycystic KD (PKD), and CKD of unknown origin. Echocardiographic measures included left ventricular (LV) ejection fraction, global longitudinal, area, and radial strain, E/e’ ratio, and LV mass index. These were compared between each aetiological group and controls in unadjusted and adjusted analysis.

**Results:**

In unadjusted analysis, patients with diabetic nephropathy/renovascular KD in diabetes mellitus, had impaired LV ejection fraction (Median [IQR]: 56% [49.9,60.69] vs. 60.8% [57.7,64.1]), global longitudinal (mean ± SD: 13.1 ± 3.5% vs. 15.5 ± 2.6%), area (24.1 ± 5.8% vs. 28.5 ± 4.2%), and radial strain (36.2 ± 11.2% vs. 44.1 ± 9.7%), and increased LV mass index (89.1 g/m^2^ [71.8,104.9] vs. 69,0 g/m^2^ [57.9,80.8]) and E/e’ ratio (10.6 [8.5,12.6] vs. 7 [5.8,8.3], p < 0.001 for all) compared with controls. Associations were similar for CKD of unknown origin. Patients with hypertensive/renovascular nephropathy had impaired global longitudinal and area strain, and higher E/e’ ratio. Patients with glomerulonephritis/vasculitis had higher LV mass index, while patients with PKD had better global longitudinal strain than controls. All findings remained significant in adjusted analysis, except for the impaired global longitudinal strain in hypertensive/renovascular nephropathy.

**Conclusion:**

Glomerulonephritis/vasculitis, hypertensive/renovascular nephropathy, CKD of unknown origin, and diabetic nephropathy/renovascular KD in diabetes mellitus were increasingly associated with adverse cardiac findings, while PKD and tubulointerstitial nephritis were not. Aetiology might play a role regarding the cardiac manifestations of CKD.

**Graphical Abstract:**

A graphical summary of the study population and main results. Abbreviations: DN = Diabetic nephropathy and renovascular kidney disease in diabetes mellitus, PKD = Polycystic kidney disease, CKDu = Chronic kidney disease of unknown origin, LVEF = Left ventricular ejection fraction, LVMi = Left ventricular mass index, E/e’ ratio = Early mitral inflow velocity to mitral annular early diastolic velocity ratio, GLS = Global longitudinal strain, GAS = Global area strain, GRS = Global radial strain.

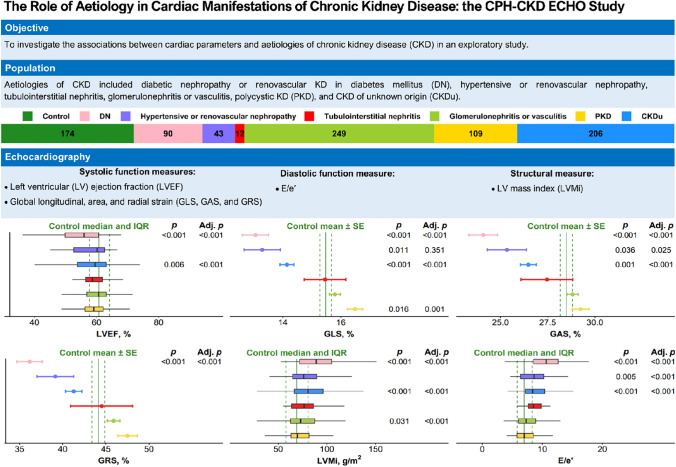

**Supplementary Information:**

The online version contains supplementary material available at 10.1007/s10554-024-03092-0.

## Introduction

The comorbidity of cardiovascular disease (CVD) in relation to chronic kidney disease (CKD) is alarmingly high [1], as it currently constitutes the most common cause of death in the patient group [2]. Hence, the KDIGO guidelines recommend that all patients with CKD be considered at increased risk of CVD [3]. The pathophysiology behind the development of CVD concomitant with CKD is suspected to involve disturbances of water and electrolyte homeostasis, sympathetic and renin–angiotensin–aldosterone-system activation, anemia, accumulation of toxins, disturbances of bone mineral and lipid metabolism, inflammation and oxidative stress [4]. The disease mechanisms thus seem to involve multiple facets of kidney disease and might vary according to the specific underlying aetiology. While some chronic kidney diseases involve aetiologies localized to the kidneys, others arise from systemic afflictions, such as diabetes mellitus or arterial hypertension. These aetiologies lead to CKD indirectly through general vascular damage with possible involvement of the entire circulatory system. This cardiovascular involvement of some aetiologies complicates investigation of the cardiac effects of CKD, as it is difficult to discern the extent of findings attributable to impaired renal function rather than to systemic effects of the underlying cause. As such, it might be of use to stratify study populations according to aetiology when investigating the association between CKD and measures of cardiac structure and function. Echocardiography is a non-invasive, low-cost method used to quantify such cardiac measures. It has been utilized in prior studies of the cardiac implications of CKD [5], but no studies exist comparing the effects of different aetiologies of CKD on cardiac structure and function. Hence, in this exploratory study, we sought to compare echocardiographic measures between different aetiologies of CKD and a healthy control population, with the aim of examining whether some aetiologies of CKD are associated with more adverse cardiac manifestations than others.

## Methods

### Population

**Fig. 1:**
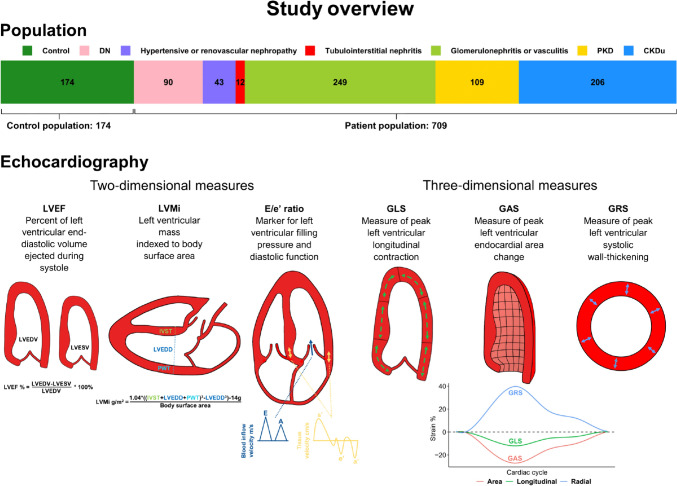
Study overview. An overview of the study population to illustrate the distribution and sample sizes of the different aetiologies of CKD. In addition, a graphical illustration of the echocardiographic measures included in the report. Abbreviations: *DN* Diabetic nephropathy and renovascular kidney disease in diabetes mellitus, *PKD* Polycystic kidney disease, *CKDu* Chronic kidney disease of unknown origin, *LVEF* Left ventricular ejection fraction, *LVMi* Left ventricular mass index, *E/e’ ratio* Early mitral inflow velocity to mitral annular early diastolic velocity ratio, *GLS* Global longitudinal strain, *GAS* Global area strain, *GRS* Global radial strain, *LVEDV* Left ventricular end-diastolic volume, *LVESV* Left ventricular end-systolic volume, *IVST* Interventricular septum thickness, *LVEDD* Left ventricular end-diastolic diameter, *PWT* Posterior wall thickness

The present report is a cross-sectional, dual-centre cohort study of outpatients with CKD, and is part of the CPH-CKD ECHO study, which has previously been described in detail [6, 7]. The original patient population consisted of 825 patients, of whom 116 were excluded from this study due to their rare aetiologies of CKD. These aetiologies included rare hereditary kidney disease, congenital malformation, and toxic kidney damage. Thus, the final patient population consisted of 709 outpatients between 26 and 76 years of age and with eGFR stages 1 to 5, not receiving dialysis treatment, and with aetiologies including diabetic nephropathy or renovascular disease in diabetes mellitus (DN, n = 90), hypertensive or renovascular nephropathy (n = 43), tubulointerstitial nephritis (n = 12), glomerulonephritis or vasculitis (n = 249), polycystic kidney disease (PKD, n = 109), and CKD of unknown origin (CKDu, n = 206). The aetiology of patients was established either through kidney biopsy if indicated and possible or through the medical history and other tests, through which a clear cause of kidney disease could be confidently established. The CKDu group consisted of patients in whom no clear cause of kidney disease could be discerned, or patients with stable CKD in which kidney biopsy was not indicated or possible. This group likely consisted of different aetiologies including hypertensive or renovascular nephropathy, tubulointerstitial nephritis, and glomerulonephritis or vasculitis among others. An overview of the population is available in Fig. 1**.** The control population consisted of 174 individuals, and the inclusion process has been described in detail elsewhere [8]. In brief, controls were matched by age and sex to patients, and excluded if kidney disease, reduced kidney function, or other chronic disease was present. Patients were consecutively included from the Departments of Nephrology, Copenhagen University Hospitals Rigshospitalet and Herlev & Gentofte Hospital, respectively, between September 2015 and August 2018. Common exclusion criteria included kidney transplantation, active malignant disease, pregnancy, mental illness, and lack of consent or ability to consent.

### Clinical characteristics

Following informed consent from individuals, investigators collected baseline data through interview, clinical examinations, and review of medical records. Clinical examinations included measurement of resting blood pressure, height, and weight. Hypertension was defined as systolic blood pressure ≥ 140 mmHg, diastolic blood pressure ≥ 90 mmHg, or use of antihypertensive medication. Participants also provided urine and blood samples, which were used for routine analysis including urine albumin-creatinine ratio (UACR) and plasma creatinine. The estimated glomerular filtration rate (eGFR) was derived from the CKD-EPI equation [9]. Review of medical records provided information regarding prevalence of diabetes and CVD. The latter was defined as suffering from one or more of the following: heart failure, previous myocardial infarction, chronic ischemic heart disease, cerebrovascular disease, or peripheral artery disease. A clinical diagnosis in the medical record was required for a CVD to be registered in the study.

### Echocardiography

The echocardiographic methods have been described in detail elsewhere [7]. In brief, echocardiography was performed by experienced investigators with GE Vingmed Ultrasound’s Vivid E9 (Horten, Norway). Subsequent analysis of the two- and three-dimensional echocardiograms, respectively, was performed by two investigators, who were blinded to all clinical information. In this study, left ventricular (LV) systolic function was assessed by LV ejection fraction (LVEF), peak global longitudinal strain (GLS), peak global radial strain (GRS), and peak global area strain (GAS). LVEF was derived from two-dimensional echocardiography and Simpson’s biplane method, while strain values were derived from three-dimensional deformation analysis. All strain values in this report are presented as absolute values. LV diastolic function was assessed by early diastolic mitral inflow velocity to early diastolic mitral annulus velocity ratio (E/e' ratio), and LV mass was assessed by LVMi, which was calculated using the Devereux method [10]. All echocardiographic parameters are illustrated graphically in Fig. 1.

### Statistical analysis

Clinical and demographic parameters were compared across all patient groups. Distribution of continuous variables was assessed using QQ-plots. Gaussian distributed continuous variables were compared with analysis of variance, and results were reported as the mean and standard deviation. Non-Gaussian distributed variables were compared using Kruskal–Wallis tests, and results were reported as the median with interquartile range. Categorical variables were compared using Pearson’s Chi^2^ tests, and results were reported as the total number with percentage. Unadjusted echocardiographic parameters were compared between each patient group and the control population using Student’s t-tests or Wilcoxon rank sum tests with Bonferroni correction. In adjusted analysis, the echocardiographic parameters were fitted using linear regression models, which were used to adjust for confounding factors including sex, age, hypertension, BMI, diabetes mellitus, smoking status, heart rate, eGFR, log_2_-transformed UACR, and CVD as defined in the “Clinical characteristics” section. These fitted values were then compared between controls and each aetiology group using Student’s t-tests or Wilcoxon rank sum tests and subsequent Bonferroni correction as in the unadjusted analysis. In addition, patient groups were also stratified according to eGFR into stage 1 + 2 (eGFR > 60 mL/min/1.73m^2^), stage 3 (eGFR 30–60 mL/min/1.73m^2)^, and stage 4 + 5 (eGFR < 30 mL/min/1.73m^2^), and according to albuminuria into A1–A3 (urine albumin-creatinine ratio of < 30 mg/g, 30-300 mg/g, and > 300 mg/g, respectively). The purpose of this stratification was to explore whether associations between aetiology and echocardiographic parameters changed with the severity of kidney disease. Due to the high prevalence of small sample sizes within subgroups produced by this stratification, no statistical testing was performed across subgroups. Results should therefore be seen as exploratory. All statistical analysis was performed using R for Windows, version 4.0.3 (R Core Team (2021). R: A language and environment for statistical computing. R Foundation for Statistical Computing, Vienna, Austria). A significance level of α = 0.05 was chosen.

## Results

Clinical and demographical data are presented in Table 1**,** and an overview of the study population and the main results can be seen in the **graphical abstract**.

**Table 1:** Baseline characteristics according to aetiology of kidney disease or control status

	Control	DN	Hypertensive or renovascular nephropathy	tubulointerstitial nephritis	Glomerulonephritis or vasculitis	PKD	CKDu	*p*
n	174	90	43	12	249	109	206	
Male sex, n (%)	107 (61.5)	62 (68.9)	26 (60.5)	8 (66.7)	162 (65.1)	59 (54.1)	142 (68.9)	0.2
Age, years, mean ± SD	59.3 ± 12.2	62.5 ± 9.1	58.7 ± 13.3	59.6 ± 11.5	53.9 ± 13.2	51.5 ± 12.1	62.6 ± 10.8	< 0.001
Smoking status, n (%)								0.2
- Never	88 (50.6)	40 (44.4)	16 (37.2)	4 (33.3)	104 (41.8)	43 (39.4)	75 (36.4)	
- Former	68 (39.1)	35 (38.9)	16 (37.2)	6 (50.0)	102 (41.0)	48 (44.0)	0 (0.0)	
- Active	18 (10.3)	15 (16.7)	10 (23.3)	2 (16.7)	42 (16.9)	18 (16.5)	40 (19.4)	
Alcohol consumption, drinks/week, median [IQR]	7 [3, 14]	1 [0, 7.5]	2 [0, 6]	1.5 [0, 10]	2 [0, 7]	2 [1, 7]	2 [0, 10]	< 0.001
Physical activity level, n (%)								< 0.001
- Low	10 (5.7)	25 (27.8)	10 (23.3)	1 (8.3)	21 (8.4)	6 (5.5)	33 (16)	
- Moderate	30 (17.2)	22 (24.4)	12 (27.9)	4 (33.3)	57 (22.9)	20 (18.3)	58 (28.2)	
- High moderate	93 (53.4)	36 (40.0)	19 (44.2)	5 (41.7)	129 (51.8)	65 (59.6)	94 (45.6)	
- High	41 (23.6)	7 (7.8)	2 (4.7)	2 (16.7)	42 (16.9)	18 (16.5)	21 (10.2)	
Heart rate, BPM, mean ± SD	70.2 ± 11.3	72.8 ± 12.8	73.4 ± 15.4	75.1 ± 9.6	74.1 ± 13.3	69.1 ± 12.3	71.7 ± 13.5	0.01
Hypertension, n (%)	56 (32.2)	80 (88.9)	41 (95.3)	9 (75.0)	202 (81.1)	94 (86.2)	180 (87.4)	< 0.001
Systolic BP, mmHg, mean ± SD	130.0 ± 16.9	136.6 ± 20.9	132.3 ± 19.5	121.1 ± 15.7)	129.7 ± 16.3	130.6 ± 15.6	134.6 ± 17.6	0.003
Diastolic BP, mmHg, mean ± SD	81.7 ± 8.9	75.2 ± 11.9	81.6 ± 11.3	75.9 ± 10.5	81.5 ± 9.7	84.0 ± 9.4	80.8 ± 11.3	< 0.001
BMI, kg/m^2^, mean ± SD	25.3 ± 3.3	32.2 ± 6.9	28.9 ± 5.9	30.8 ± 7.7	27.9 ± 5.6	26.7 ± 4.7	29.1 ± 5.9	< 0.001
Diabetes, n (%)	0 (0)	90 (100)	10 (23.3)	4 (33.3)	23 (9.2)	5 (4.6)	28 (13.6)	< 0.001
HbA1c, median [IQR]	35 [32, 36]	54 [47, 65]	38 [35, 43]	41 [36, 43]	37 [34, 40]	35 [33, 38]	37 [35, 42]	< 0.001
Total plasma cholesterol, mmol/L, mean ± SD	5.6 ± 1.0	4.3 ± 1.1	5.0 ± 1.1	5.1 ± 1.7	5.6 ± 1.3	5.1 ± 1.0	4.9 ± 1.1	< 0.001
Cardiovascular disease, n (%)	0 (0)	39 (43.3)	8 (18.6)	1 (8.3)	5 (2.0)	1 (0.9)	56 (27.2)	< 0.001
Heart failure, n (%)	0 (0)	24 (26.7)	8 (18.6)	1 (8.3)	5 (2)	1 (0.9)	34 (10.6)	< 0.001
Previous myocardial infarction, n (%)	0 (0)	9 (10.0)	3 (7.0)	1 (8.3)	7 (2.8)	1 (0.9)	12 (5.8)	0.005
Significant valve disease, n (%)	0 (0)	10 (11.1)	1 (2.3)	0 (0.0)	4 (1.6)	1 (0.9)	16 (5.0)	< 0.001
Previous stroke, n (%)	0 (0)	18 (20.0)	6 (14.0)	0 (0.0)	11 (4.4)	6 (5.5)	41 (12.7)	< 0.001
Peripheral artery disease, n (%)	0 (0)	10 (11.1)	8 (18.6)	1 (8.3)	3 (1.2)	1 (0.9)	16 (5.0)	< 0.001
Kidney biopsy, yes, n (%)	0 (0)	10 (11.1)	12 (27.9)	6 (50.0)	78 (31.3)	0 (0.0)	0 (0.0)	< 0.001
eGFR, median [IQR]	81 [73, 91]	35 [22.2, 41.8]	31 [22, 44.5]	26.5 [17.2, 34.8]	51 [36, 77]	54 [24, 79]	39.5 [27, 53]	< 0.001
eGFR stage, n (%)								< 0.001
- Control	174 (0)	0 (0.0)	0 (0.0)	0 (0.0)	0 (0.0)	0 (0.0)	0 (0.0)	
- Stage 1	0 (0)	1 (1.1)	0 (0.0)	0 (0.0)	39 (15.7)	19 (17.4)	16 (5.0)	
- Stage 2	0 (0)	4 (4.4)	1 (2.3)	0 (0.0)	63 (25.3)	28 (25.7)	41 (12.7)	
- Stage 3a	0 (0)	14 (15.6)	10 (23.3)	2 (16.7)	49 (19.7)	17 (15.6)	66 (20.5)	
- Stage 3b	0 (0)	35 (38.9)	12 (27.9)	4 (33.3)	58 (23.3)	12 (11.0)	106 (32.9)	
- Stage 4	0 (0)	27 (30.0)	16 (37.2)	4 (33.3)	36 (14.5)	20 (18.3)	76 (23.6)	
- Stage 5	0 (0)	9 (10.0)	4 (9.3)	2 (16.7)	4 (1.6)	13 (11.9)	17 (5.3)	
UACR, median [IQR]	2 [2, 4]	245.5 [39, 1,514.8]	161 [12, 1,036]	46 [5.5, 177]	327 [55, 922]	26 [13, 90]	101 [16, 453]	< 0.001
Albuminuria status, mg/g, n (%)								< 0.001
- None or mild	173 (99.4)	25 (27.8)	15 (34.9)	6 (50.0)	48 (19.3)	62 (56.9)	120 (37.3)	
- Moderate	1 (0.6)	25 (27.8)	11 (25.6)	4 (33.3)	75 (30.1)	34 (31.2)	103 (32.0)	
- Severe	0 (0)	40 (44.4)	17 (39.5)	2 (16.7)	126 (50.6)	13 (11.9)	99 (30.7)	

*SD* Standard deviation, *IQR* Interquartile range, *BPM* Beats per minute, *BP* Blood pressure, *BMI* Body mass index, *eGFR* estimated glomerular filtration rate, *UACR* Urine albumin-creatinine ratio, *DN* Diabetic nephropathy or renovascular kidney disease in diabetes mellitus, *PKD* Polycystic kidney disease, *CKDu* Chronic kidney disease of unknown origin

In summary, patients with DN had the highest systolic blood pressure, BMI, HbA1c, prevalence of diabetes mellitus, and lowest diastolic blood pressure, physical activity level, and total plasma cholesterol level. Patients with DN and CKDu were the oldest, while patients with PKD and glomerulonephritis or vasculitis groups were the youngest. With regards to cardiovascular comorbidities, the DN group was associated with the highest prevalence of CVD, including heart failure, previous acute myocardial infarction, and significant valve disease. Prevalence of hypertension was much lower in the control group compared with all disease groups. The hypertensive or renovascular nephropathy group had the highest prevalence of hypertension, followed by the DN and CKDu groups. With regards to renal parameters, glomerulonephritis or vasculitis was associated with the highest UACR, while tubulointerstitial nephritis was associated with the lowest eGFR. Controls were generally more physically active, smoked less, and had higher alcohol consumption than the patient population. The number of cases confirmed through kidney biopsy differed between groups, with tubulointerstitial nephritis, glomerulonephritis or vasculitis, and hypertensive or renovascular nephropathy being the most prevalently biopsied, while no cases of CKDu or PKD were verified through biopsy.

### Echocardiographic findings

Echocardiographic parameters for each patient group were compared with the control population, and significant findings are listed in Table 2.

**Table 2:** Unadjusted and adjusted comparison of echocardiographic parameters between controls and individual patient groups. Only patient groups which differed significantly from controls in unadjusted analysis were included. Adjusted analysis was performed using multivariable linear regression to account for potential confounding

	Control	CKD	*p*	Fitted^a^ control	Fitted^a^ CKD	Adj.* p*^b^
*LVEF, %, median [IQR]*						
DN	60.8 [57.7, 64.1]	56.0 [49.9, 60.6]	< 0.001	60.7 [60, 61.8]	54.2 [51.7, 55.7]	< 0.001
CKDu	60.8 [57.7, 64.1]	59.5 [53.8,63.1]	0.006	60.7 [60, 61.8]	58.8 [55.8, 59.9]	< 0.001
*GLS, %, mean ± SD*						
DN	15.5 ± 2.6	13.1 ± 3.5	< 0.001	15.6 ± 1.1	13.6 ± 1.3	< 0.001
Hypertensive or renovascular nephropathy	15.5 ± 2.6	13.3 ± 3.8	0.011	15.6 ± 1.1	15 ± 2.2	0.351
CKDu	15.5 ± 2.6	14.1 ± 3	< 0.001	15.6 ± 1.1	14.8 ± 1.4	< 0.001
PKD	15.5 ± 2.6	16.5 ± 2.7	0.016	15.6 ± 1.1	16.2 ± 1.4	0.001
*GAS, %, mean ± SD*						
DN	28.5 ± 4.2	24.1 ± 5.8	< 0.001	28.6 ± 1.4	24.9 ± 2	< 0.001
Hypertensive or renovascular nephropathy	28.5 ± 4.2	25.3 ± 6.2	0.036	28.6 ± 1.4	26.9 ± 2.7	0.025
CKDu	28.5 ± 4.2	26.5 ± 5.2	0.001	28.6 ± 1.4	27.3 ± 2	< 0.001
*GRS, %, mean ± SD*						
DN	44.1 ± 9.7	36.2 ± 11.2	< 0.001	44.6 ± 3.1	38.8 ± 4.2	< 0.001
*LVMi, g/m*^*2*^*, median [IQR]*						
DN	69 [57.9, 80.8]	89.1 [71.8, 104.9]	< 0.001	69.5 [62.6, 76.4]	92.7 [86.5, 97.8]	< 0.001
CKDu	69 [57.9, 80.8]	80.6 [66.3,96.1]	< 0.001	69.5 [62.6, 76.4]	82.4 [75.1, 88.6]	< 0.001
Glomerulonephritis or vasculitis	69 [57.9, 80.8]	72.8 [62.6, 87.6]	0.031	69.5 [62.6, 76.4]	77.8 [70.1, 83.7]	< 0.001
*E/e’ ratio, median [IQR]*						
DN	7 [5.8, 8.3]	10.6 [8.5, 12.6]	< 0.001	7.5 [6.8, 7.9]	11.4 [10.1, 12.3]	< 0.001
Hypertensive or renovascular nephropathy	7 [5.8, 8.3]	8.6 [6.4, 10.2]	0.005	7.5 [6.8, 7.9]	8.9 [7.5, 10.4]	< 0.001
CKDu	7 [5.8, 8.3]	8.4 [7.2,10.4]	< 0.001	7.5 [6.8, 7.9]	8.8 [7.9, 10.2]	< 0.001

*CKD* Chronic kidney disease, *LVEF* Left ventricular ejection fraction, *IQR* Interquartile range, *DN* Diabetic nephropathy or renovascular kidney disease in diabetes mellitus, *CKDu* Chronic kidney disease of unknown origin, *GLS* Global longitudinal strain, *SD* Standard deviation, *PKD* Polycystic kidney disease, *GAS* Global area strain, *GRS* Global radial strain, *LVMi* Left ventricular mass index, *E/e’ ratio* Early mitral inflow velocity to mitral annular early diastolic velocity ratio

^a^Adjusted for sex, age, BMI, diabetes mellitus, smoking status, hypertension, heart rate, cardiovascular disease, eGFR, and log_2_-transformed UACR

^b^Derived from student’s t-tests or Wilcoxon ranks sum tests with subsequent Bonferroni correction performed on fitted values

**Fig. 2:**
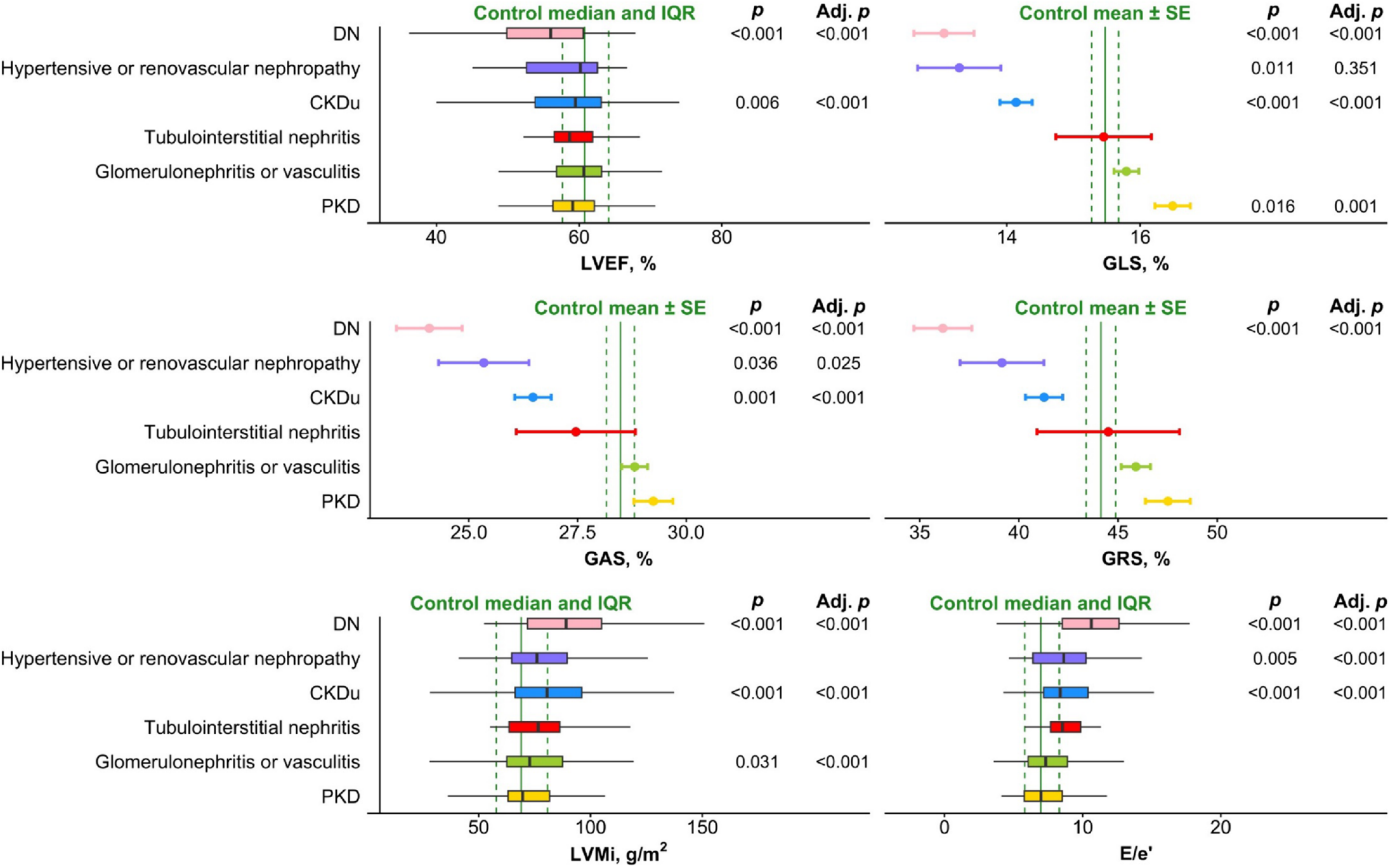
Echocardiographic parameters according to aetiology. Dots represent mean values, and error bars represent standard error, while boxplots represent medians and quantiles. The solid vertical line in each graph represents the mean or median value of the parameter in the control population, and the dashed lines represent standard error or interquartile range, respectively. The *p*-values are derived from Student’s t-tests or Wilcoxon ranks sum tests with subsequent Bonferroni correction performed between each group and the control population. The adjusted *p*-values are derived from Student’s t-tests or Wilcoxon ranks sum tests with subsequent Bonferroni correction performed on fitted values derived from multivariable linear regressions adjusting for sex, age, BMI, diabetes mellitus, smoking status, hypertension, heart rate, cardiovascular disease, eGFR and log_2_-transformed UACR. Abbreviations: *DN* Diabetic nephropathy and renovascular kidney disease in diabetes mellitus, *CKDu* Chronic kidney disease of unknown origin, *PKD* Polycystic kidney disease, *Adj*. *p* Adjusted p-value, *IQR* Interquartile range, *SE* Standard error, *LVEF* Left ventricular ejection fraction, *GLS* Global longitudinal strain, *GAS* Global area strain, *GRS* Global radial strain, *LVMi* Left ventricular mass index, *E/e’ ratio* Early mitral inflow velocity to mitral annular early diastolic velocity ratio

Results from unadjusted and adjusted analysis are also illustrated in Fig. 2.

**Fig. 3:**
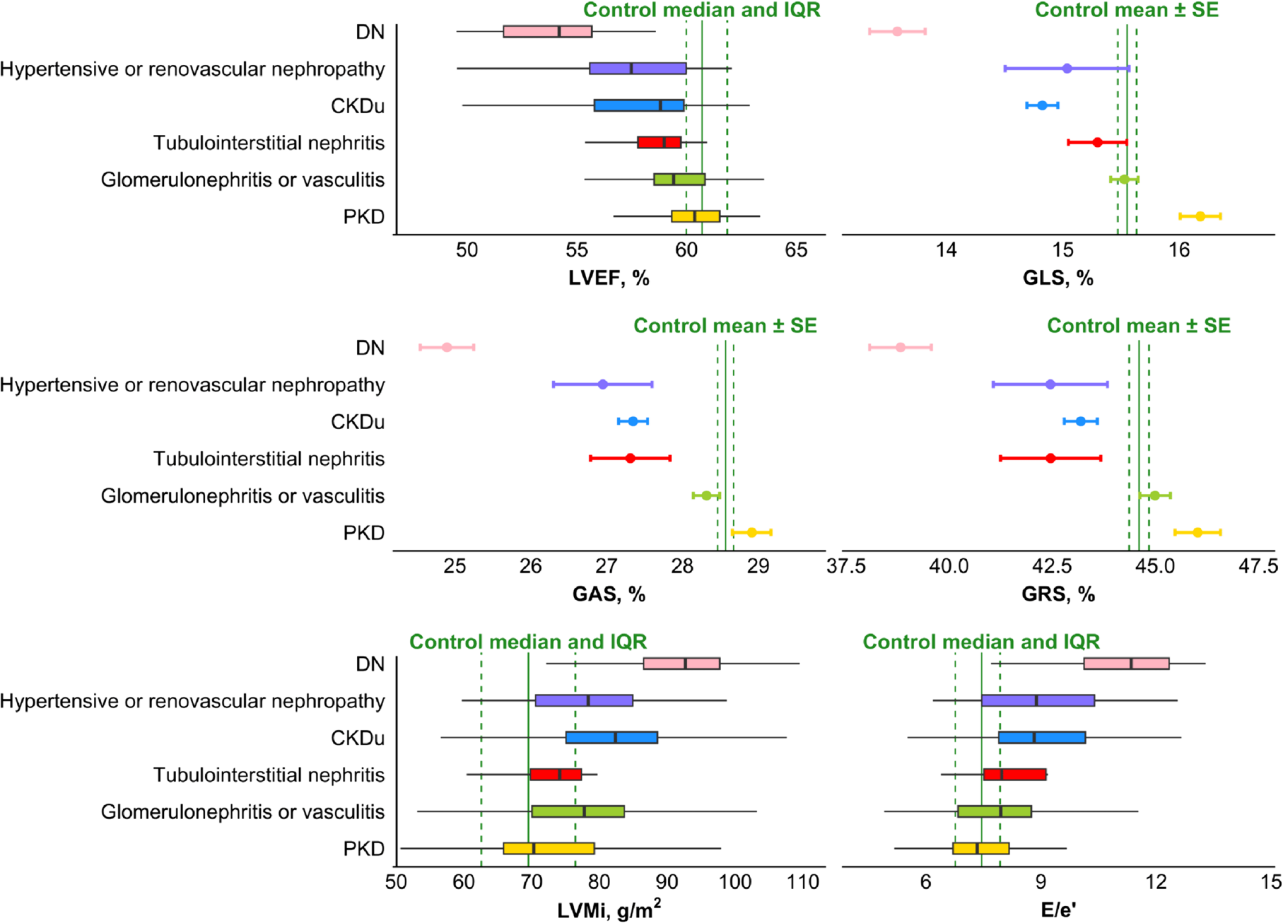
Adjusted echocardiographic parameters according to aetiology. Dots represent mean values, and error bars represent standard error, while boxplots represent medians and quantiles. The solid vertical line in each graph represents the mean or median value of the parameter in the control population, and the dashed lines represent standard error or interquartile range, respectively. The adjusted values presented are derived from multivariable linear regressions adjusting for sex, age, BMI, diabetes mellitus, smoking status, hypertension, heart rate, cardiovascular disease, eGFR and log_2_-transformed UACR. Abbreviations: *DN* Diabetic nephropathy and renovascular kidney disease in diabetes mellitus, *CKDu* Chronic kidney disease of unknown origin, *PKD* Polycystic kidney disease, *Adj. p* Adjusted p-value, *IQR* Interquartile range, *SE* Standard error, *LVEF* Left ventricular ejection fraction, *GLS* Global longitudinal strain, *GAS* Global area strain, *GRS* Global radial strain, *LVMi* Left ventricular mass index, *E/e’ ratio* Early mitral inflow velocity to mitral annular early diastolic velocity ratio

In unadjusted analysis, DN was associated with reduced LVEF, GLS, GAS, and GRS. It was further associated with increased LVMi and E/e’ ratio. CKDu was similarly associated with reduced LVEF, GLS, GAS, and GRS, and with increased LVMi and E/e’ ratio. Hypertensive or renovascular nephropathy was associated with reduced GLS and GAS, and further with increased E/e’ ratio. Lastly, PKD was associated with increased GLS, while glomerulonephritis or vasculitis was associated with increased LVMi. In adjusted analysis (Table 2), all findings remained significant, except for reduced GLS in the hypertensive or renovascular nephropathy group. All adjusted values can be seen in Fig. 3.

**Fig. 4:**
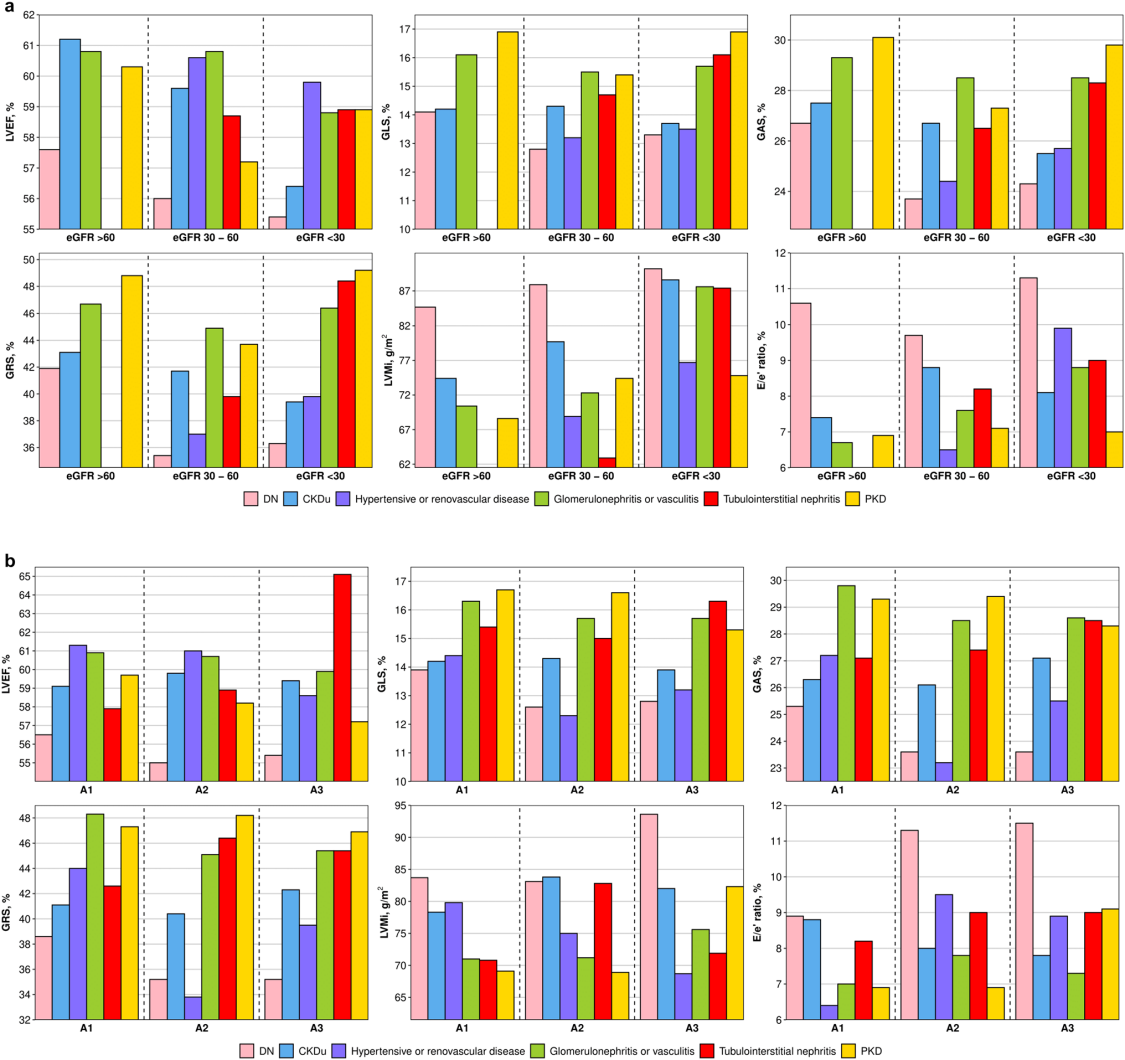
**a** and **b** Echocardiographic parameters according to aetiology stratified by renal function. Bar plots illustrating echocardiographic parameters according to aetiology when further stratified according to renal function as assessed by eGFR stage and severity of albuminuria. Subjects were divided according to eGFR groups corresponding to CKD stage 1 + 2, stage 3, and stage 4 + 5, respectively. Albuminuria stages A1-3 correspond to a urine albumin-creatinine ratio of < 30 mg/g, 30–300 mg/g, and > 300 mg/g, respectively. Number of subjects per group can be seen in Table 3. Only one subject was included in the hypertensive or renovascular group in CKD stage 1 + 2 and was therefore not included in** a**. *DN* Diabetic nephropathy and renovascular kidney disease in diabetes mellitus, *CKDu* Chronic kidney disease of unknown origin, *PKD* Polycystic kidney disease, *eGFR* estimated glomerular filtration rate, *LVEF* Left ventricular ejection fraction, *GLS* Global longitudinal strain, *GAS* Global area strain, *GRS* Global radial strain, *LVMi* Left ventricular mass index, *E/e’ ratio* Early mitral inflow velocity to mitral annular early diastolic velocity ratio

The unadjusted echocardiographic findings for all groups can be found in supplemental Table 1. When groups were further stratified according to eGFR stage and albuminuria severity, respectively, all measures for DN were consistently impaired across all eGFR strata compared with other aetiologies (Fig. 4a).

The same was largely true across albuminuria strata with only hypertensive or renovascular nephropathy having more impaired strain parameters in A2, and CKDu having higher LVMi in A2 (Fig. 4b). Number of subjects per subgroup can be seen in Table 3**.**

**Table 3:** eGFR stages and albuminuria severity according to aetiology of CKD

Group	eGFR			Albuminuria		
	Stage 1 and 2	Stage 3	Stage 4 and 5	A1	A2	A3
Diabetic nephropathy	5	49	36	25	25	40
Glomerulonephritis or vasculitis	34	112	60	48	75	126
Hypertensive/renovascular nephropathy	1	22	20	15	11	17
CKD of unknown origin	102	107	40	83	64	59
Polycystic kidney disease	47	29	33	62	34	13
Tubulointerstitial nephropathy	0	6	6	6	4	2

## Discussion

To the best of our knowledge, the present report constitutes the first study comparing echocardiographic parameters between populations with different CKD aetiologies and a healthy control population. The findings of this large-scale cohort study include significantly impaired diastolic and systolic function, and increased LV mass associated with DN and CKDu compared with healthy controls. Hypertensive or renovascular nephropathy was associated with impaired systolic and diastolic function, while glomerulonephritis or vasculitis was associated with slightly increased LV mass. In contrast, PKD was associated with better systolic function than the control population. Thus, tubulointerstitial nephritis and PKD showed no adverse associations, while glomerulonephritis or vasculitis was only associated with slight structural differences. Conversely, DN, CKDu and hypertensive or renovascular nephropathy were associated with adverse findings in all or most of the examined measures of cardiac structure and function.

### CKD caused by systemic disease

Several aetiologies discussed in this report are results of systemic underlying diseases, such as diabetes mellitus or hypertension. Myocardial dysfunction is a common finding in relation to diabetes mellitus [11, 12]. The systemic effects of diabetes mellitus are believed to result from hemodynamic, metabolic, and inflammatory abnormalities leading to vascular complications, including atherosclerosis, retinopathy, nephropathy, and likely also myocardial dysfunction [13, 14]. A previous echocardiographic study found increased LVMi and decreased LV systolic shortening in patients with DN compared with normoalbuminuric patients [15], suggesting increased cardiovascular effects of diabetes mellitus with renal involvement. We believe that there are three possible explanations for the increased extent of these cardiac effects in DN compared with diabetes mellitus. The first possibility is the additive cardiovascular effects of diabetes mellitus coupled with the known adverse effects of declining renal function. The second possibility is that diabetes mellitus drives both the renal and cardiac insult, and the renal involvement thus is a passive comorbidity of CVD rather than a cause of it. The third and most likely explanation is a combination of the first two, as the kidneys are intrinsically linked with the cardiovascular system. Similar to diabetes, hypertension is a common cause of both CKD and CVD. Additionally, increasing renal involvement in hypertension, assessed by UACR, has also been linked with increasingly impaired LV diastolic and systolic function, and with increasing LVMi [16, 17]. In this study, we found associations between hypertensive or renovascular nephropathy, and decreased LV diastolic and systolic function. As with DN, it is difficult to establish the extent to which the adverse cardiac findings in hypertensive nephropathy can be explained by the underlying affliction, hypertension. In continuation of this, it is of interest that even though the prevalence of hypertension in the PKD group was almost as high as that of the hypertensive or renovascular nephropathy group, LVMi in patients with PKD was the lowest amongst patients. This might be due to earlier onset of antihypertensive treatment within this patient group, while individuals from the hypertensive or renovascular nephropathy group might have been afflicted with undetected or uncontrolled hypertension for longer periods of time leading to renal and perhaps also cardiac damage.

CKDu also showed similar associations with DN. Since this group likely contains cases of both DN and hypertensive or renovascular nephropathy, which were not verified through renal biopsy, the similarities in results between CKDu and these groups might be explained by common aetiologies.

Future longitudinal studies following the development and progress of adverse cardiac manifestations in patients with DN and hypertensive or renovascular nephropathy are warranted to elucidate the respective roles of the underlying systemic diseases and the consequential renal involvement. Furthermore, it could be of interest for future studies to take into account the severity and duration of the underlying disease.

### Cardiac abnormalities in CKD and the role of albuminuria and eGFR

This study found that PKD and tubulointerstitial nephritis were not associated with adverse cardiac findings compared with controls. Additionally, the study found only slightly increased LV mass in patients with glomerulonephritis or vasculitis. This is interesting for two main reasons. Firstly, because it is well established, that CKD is associated with numerous cardiac abnormalities, including LV hypertrophy and reduced LV diastolic and systolic function [18–21]. Our findings could suggest that these adverse associations are, at least in part, dependent upon the underlying aetiologies in addition to the impaired renal function.

Secondly, it is interesting because markers of renal function, eGFR and albuminuria, are also directly correlated with adverse cardiac alterations in CKD [7, 18, 22, 23]. Albuminuria has been linked with impaired systolic function in different populations, including in heart failure with preserved ejection fraction [24], and in type 1 diabetes mellitus [12]. In this study, patients with glomerulonephritis or vasculitis exhibited the highest prevalence of severe albuminuria, but did not differ significantly from the control group in LV function. This could indicate that the context in which albuminuria occurs might influence the association with cardiac structure and function. While not conclusive evidence, this might serve as a potential hypothesis for future studies. Similarly, tubulointerstitial nephritis was associated with the lowest eGFR, but did not differ from the control group with regards to cardiac structure or function either, albeit this finding is weakened by the small sample size of the patient group.

### Clinical perspective

Compared with controls, we found that DN and hypertensive or renovascular nephropathy were related to more adverse cardiac findings than other aetiologies of CKD, even after adjustment for traditional risk factors and eGFR and albuminuria. Contrarily, glomerulonephritis/vasculitis, tubulointerstitial nephritis, and especially PKD did not differ significantly from healthy controls. Several of the echocardiographic parameters addressed in this report are prognostic of adverse outcomes in the general population and in populations with CKD, including cardiovascular morbidity and mortality [22, 25–27]. While eGFR and albuminuria are often regarded in relation to the assessment of cardiovascular risk of patients with CKD, the findings of this study might indicate a potential prognostic benefit of further considering aetiology in addition to eGFR and albuminuria. This might be the subject of future longitudinal works.

### Limitations

The results of this study should be interpreted within the context of its limitations. These include the unequal sample sizes of the patient groups, which may have influenced the results of statistical analyses. In addition, the aetiology was not confirmed through biopsy in all cases, and the prevalence of biopsy-verified cases differed between groups, in part also due to less relevance with some aetiologies, for example PKD. Our population size unfortunately did not allow for stratification according to both aetiology, eGFR and albuminuria, which could have supported the association between certain aetiologies and adverse cardiac findings independent of the extent of kidney disease. In addition, information regarding duration of kidney disease was not available. The ethnicity of the study population was relatively uniform, and therefore not necessarily representative of all ethnicities. Similarly, due to the multiple visits and tests related to the study, the population might be slightly affected by volunteer bias. The cross-sectional design of the study precludes the possibility of establishing causality between aetiologies and echocardiographic findings, and merely serves to demonstrate associations. Thus, it is possible that these patients already exhibited impaired cardiac function or structure prior to the development or time of diagnosis of their CKD. Future longitudinal studies are warranted to monitor potential decline or alterations in cardiac function and mass concurrently with the development and progress of the specific chronic kidney diseases.

## Conclusion

Our study found that patients with glomerulonephritis or vasculitis, hypertensive or renovascular nephropathy, CKDu, and DN differed increasingly and adversely from a healthy control population in measures of LV structure and function, while patients with PKD and tubulointerstitial nephritis did not, regardless of traditional cardiovascular risk factors and markers of renal function. These results should be regarded as hypothesis generating, and future studies are warranted to investigate whether aetiology affects the cardiac manifestations of chronic kidney disease.

### Supplementary Information

Below is the link to the electronic supplementary material.

Supplementary file1 (DOCX 17 KB)
