# Supplementary material for: The role of aetiology in cardiac manifestations of chronic kidney disease: the CPH-CKD ECHO study
Source: Int J Cardiovasc Imaging. 2024 Apr 30;40(6):1221–33. doi: 10.1007/s10554-024-03092-0 (PMC11213755; doi:10.1007/s10554-024-03092-0)
Supplement: Supplementary file 1 — Supplementary file1 (DOCX 17 KB) [file 10554_2024_3092_MOESM1_ESM.docx]

|  | **Control** | **DN** | **Hypertensive or renovascular nephropathy** | **Tubulointerstitial nephritis** | **Glomerulonephritis or vasculitis** | **PKD** | **CKDu** | ***p*** |
| --- | --- | --- | --- | --- | --- | --- | --- | --- |
| n | 174 | 90 | 43 | 12 | 249 | 109 | 206 |  |
| LVEF, %, median [IQR] | 60.8 [57.7, 64.1] | 56.0 [49.9, 60.6] | 60.2 [52.6, 62.6] | 58.7 [56.5, 61.9] | 60.7 [56.8, 63.1] | 59.1 [56.3, 62.1] | 59.5 [53.8, 63.1] | <0.001 |
| GLS, %, mean±SD | 15.5±2.6 | 13.1±3.5 | 13.3±3.8 | 15.5±2.4 | 15.8±2.8 | 16.5±2.7 | 14.1±3.0 | <0.001 |
| GAS, %, mean±SD | 28.5±4.2 | 24.1±5.8 | 25.3±6.2 | 27.5±4.5 | 28.8±4.5 | 29.2±4.6 | 26.5±5.2 | <0.001 |
| GRS, %, mean±SD | 44.1±9.7 | 36.2±11.2 | 39.1±12.7 | 44.5±11.9 | 45.9±11.0 | 47.5±11.6 | 41.3±11.7 | <0.001 |
| LVMi, g/m^2^, median [IQR] | 69.0 [57.9, 80.8] | 89.1 [71.8, 104.9] | 76.1 [64.8, 89.6] | 76.6 [63.6, 86.2] | 72.8 [62.6, 87.6] | 69.7 [63.1, 81.7] | 80.6 [66.3, 96.1] | <0.001 |
| E/e’ ratio, median [IQR] | 7.0 [5.8, 8.3] | 10.6 [8.5, 12.6] | 8.6 [6.4, 10.2] | 8.5 [7.7, 9.8] | 7.3 [6.0, 8.9] | 7.0 [5.8, 8.5] | 8.4 [7.2, 10.4] | <0.001 |

*DN = Diabetic nephropathy and renovascular kidney disease in diabetes mellitus, PKD = Polycystic kidney disease, CKDu = Chronic kidney disease of unknown origin, LVEF = Left ventricular ejection fraction, IQR = Interquartile range, SD = Standard deviation, GLS = Global longitudinal strain, GAS = Global area strain, GRS = Global radial strain, LVMi = Left ventricular mass index, E/e’ ratio = Early mitral inflow velocity to mitral annular early diastolic velocity ratio.*

**Supplemental table 1**
